# Estimation of Benchmark Dose of Cumulative Cadmium Exposure for Renal Tubular Effect

**DOI:** 10.3390/ijerph18105177

**Published:** 2021-05-13

**Authors:** Kazuhiro Nogawa, Yasushi Suwazono, Yuuka Watanabe, Carl-Gustaf Elinder

**Affiliations:** 1 Department of Occupational and Environmental Medicine, Graduate School of Medicine, Chiba University, Chiba 260-8670, Japan; nogawa@chiba-u.jp (K.N.); watanabe155@chiba-u.jp (Y.W.); 2 Department of Clinical Science, Intervention and Technology, Karolinska Institute, 17177 Stockholm, Sweden; Carl-Gustaf.Elinder@ki.se

**Keywords:** air cadmium, benchmark dose, blood cadmium, occupational exposure

## Abstract

Objectives: The aim of this study was to determine the no observed adverse effect level (NOAEL), the lowest observed adverse effect level (LOAEL) and the benchmark dose low (BMDL) of cadmium exposure by re-evaluation of the dose–response relationship between cumulative cadmium exposure and renal tubular damage reported previously. Methods: The participants were workers (326 men and 114 women) employed for at least three months between 1931 and 1982. Blood cadmium (Cd-B) and air cadmium (Cd-A) were collected at regular intervals with urinary β2-microglobulin as the tubular effect marker. Cumulative Cd-A and Cd-B were estimated by multiplying concentration and working period. The BMDL was calculated using Benchmark Dose Software (version 3.1.2). The benchmark response (BMR) was set at 5% or 10%. Results: By logistic regression, the NOAEL of mean cumulative Cd-B was 7122 months nmol/L. The LOAEL of cumulative Cd-A and least-squares cumulative Cd-B was 691 yrs μg/m^3^ and 8586 months nmol/L, respectively. Among various models for dose–response relationships, a probit model was adopted as the best fitting model. The obtained BMDLs of cumulative Cd-A were 272.3 yrs µg/m^3^ (BMR5%) and 707.5 yrs µg/m^3^ (BMR10%). The BMDLs of mean cumulative Cd-B were 3967.2 months nmol/L (BMR5%) and 7798.1 months nmol/L (BMR10%). The BMDLs of least-squares cumulative Cd-B were 3588.6 months nmol/L (BMR5%) and 8616.3 months nmol/L (BMR10%). Assuming a working period of 40 years, the BMDLs for BMR10% corresponded to 17.7 µg/m^3^ (Cd-A) and 1.8~2.0 µg/L (Cd-B). Discussion: This study provides new valuable information to enhance the reliability of limit values and thereby make a significant contribution to preventing the health effects of Cd in exposed workers.

## 1. Introduction

Chronic respiratory exposure to cadmium (Cd) causing emphysema, renal and bone damage, and Cd-induced adverse renal effects starts with proximal tubular damage followed by glomerular damage. Bone effects are characterized by osteomalacia and osteoporosis [1,2]. Itai-Itai disease is the most severe form of chronic Cd poisoning caused by prolonged Cd exposure, and has afflicted inhabitants of the Jinzu River basin in Toyama Prefecture [1,3]. In order to prevent Cd-induced health effects in workers, it is essential to establish the reference level of Cd exposure that might raise the possibility of an adverse renal effect.

Exposure to cadmium through the inhalation of fumes or dust in occupational settings has long been known as the main cause of adverse health effects on lungs and kidneys [1]. Therefore, the air Cd concentration (Cd-A) has been thought to be an important index of Cd exposure in workers. To prevent the chronic effects of Cd such as renal damage, it is useful to determine the cumulative dose. Commonly, the individual dose is estimated based on the average level of cadmium in air at the worksite multiplied by the actual period of exposure [4,5]. In workers at a copper-Cd alloy manufacturing plant in the UK, significant correlations have been reported between the cumulative air Cd exposure index (yrs μg/m^3^), calculated as air Cd concentration × years of work, and Cd concentrations in liver, kidney, blood, and urine [5]. Furthermore, the cumulative air Cd index was significantly related to various urinary tubular and glomerular markers such as β2-microglobulin (β2-MG) [5].

In terms of blood Cd level (Cd-B), it is well-known that there are fast and slow components for the reduction of blood cadmium, reflecting recent exposure and long-term exposure, respectively [1]. After long-term cadmium exposure, an increasing proportion of blood cadmium will be related to the body burden, and blood cadmium is a good indicator of internal dose and accumulation in the kidney and other soft tissues [1]. An even better indicator of body burden would be the cumulative exposure calculated from repeated blood samples or time-integrated Cd-B [1]. Reflecting this feature, the relationship between cumulative Cd-B and urinary tubular markers has been reported in several studies [4,6,7]. To investigate this, cumulative cadmium doses were estimated individually for 440 workers in a battery factory [4]. Cumulative Cd-A as well as two cumulative Cd-B were computed for each individual. A significant correlation was observed between cumulative Cd-A and Cd-B [4]. Forty workers had evidence of renal tubular damage determined by urinary β2-MG. By applying the probit model to the data, significant dose–response relationships were observed between cumulative Cd-A or Cd-B and renal tubular damage [4].

Based on this information, the biological tolerable limits for occupational Cd exposure have been proposed as the Threshold Limit Value (TLV, 10 µg/m^3^, (total particulate) 2 µg/m^3^ (respirable particulate fraction)) [8] and Biological Exposure Index (BEI, 5 µg/L (Cd-B) and 5 µg/g cre (urinary Cd)) [9] of the American Conference of Governmental Industrial Hygienists (ACGIH) and the 8-h time weighted average (8 h-TWA, 1 µg/m^3^ (inhalable fraction)) and the Biological Exposure Values (BEV, 2 µg/g cre (urinary Cd)) of the Scientific Committee on Occupational Exposure Limits (SCOEL) of the European Union [10].

The concept of the benchmark dose (BMD), published by Crump et al., has been widely applied to the risk assessment of environmental chemicals [11,12]. This concept has been taken up by the US Environmental Protection Agency (EPA) and the Environmental Health Criteria of the World Health Organization (WHO). As an alternative to the no observed adverse effect level (NOAEL) and lowest observed adverse effect level (LOAEL), BMD Low (BMDL), which is the lower limit of the 95% confidence interval for the benchmark dose (BMD), which corresponds to a certain increase in the rate of findings (benchmark response, BMR) from the unexposed population, has been considered useful, and calculated as regarding the adverse health effects of many environmental contaminants. The US EPA recommends a BMR of 10% as the initial value for calculating BMDL (BMDL_10_) in laboratory animals; however, in the case of human epidemiological data, it may be necessary to adopt a lower BMR of 5% (BMDL_05_) or 1% [13]. However, up to now, BMDL has not been calculated for Cd-A or Cd-B in workers with occupational Cd exposure. Furthermore, few reports have revealed the NOAEL and LOAEL for Cd-A and Cd-B in the working population.

This prompted us to determine the NOAEL, LOAEL and BMDL by re-evaluation of the fine dose–response relationship between cumulative Cd exposure and renal tubular damage in previously reported reliable data [4].

## 2. Methods

### 2.1. Study Population and Exposure Assessment

As noted in the previous report [4], the participants were workers (326 men and 114 women) employed for at least three months between 1931 and 1982. Measurements of Cd-A have been conducted since 1947. Information on the length of employment for each individual studied was obtained from the company files. An individual dose based on cumulative Cd-A was calculated according to the following Equation (1):

Cum Cd-A = Sum D_i_ × C_i_, (yrs μg/m^3^) (1)

where D_i_ = years of employment at a particular level of Cd-A, C_i_ = average Cd-A during the corresponding period. Cd-B values have been collected since 1967 at regular intervals together with other laboratory data, such as urinary β2-MG, which was chosen as the tubular effect marker in this study. The mean number of blood tests per individual was 7 (±5, range 0–26). Three workers were excluded because of missing Cd-B data, and thus 437 workers were included for the cumulative blood-cadmium analyses. Initially, average Cd-B was computed from the available Cd-B values for each worker. This average value was then multiplied by the number of months employed at the factory according to Equation (2):

Mean cum Cd-B = Sum T_i_ × B_i_ (months nmol/L) (2)

where T_i_ = number of months employed, B_i_ = average Cd-B.

Considering the very high exposure in the 1940s or 1950s, this mean Cd-B may underestimate the whole cumulative exposure [4]. Thus, another cumulative dose was calculated fitting the secular change in Cd-B to a straight line using least-squares (leasq) estimation according to Equation (3):

Leasq cum Cd-B = 1/2 × T_i_ × (DO + D1) (3)

where T_i_ = number of months employed, D0 = k Y0 + I, D1 = k Y1 + I, k = slope, I = intercept for the fitted line, Y0 = year of first employment and Y1 = last year of employment or end of study, whichever comes first [4].

### 2.2. Outcome

As the renal tubular marker, creatinine-adjusted urinary β2-MG was measured using a radioimmunoassay method (Phadebas, Pharmacia, Uppsala, Sweden) from 1972 to 1982. β2-MG uria was defined as β2-MG > 35 µg/mmol cre (309.4 µg/g cre). This urinary β2-MG level was based on the upper 2.5 percentile for the urinary β2-MG among persons without tubular dysfunction [4], and was identical to the cut-offs adopted by the European Food Safety Authority (EFSA) (300 µg/g crea) [14] or the inflection point of the urinary Cd and β2-MG relationship adopted by the Joint FAO/WHO Expert Committee on Food Additives (JECFA) [15] as for the threshold of Cd exposure in the large meta-analyses. To avoid single laboratory findings above the critical level influencing the results, the urinary β2-MG values were determined by calculating an average of all the available measurements.

### 2.3. Statistical Analysis

Odds ratios of the categorized cum Cd-A, mean cum Cd-B and leasq cum Cd-B level for the β2-MG uria were evaluated using logistic regression by IBM SPSS 19J (IBM Business Analytics, Tokyo, Japan). Lowest categories were adopted as the reference one.

Furthermore, we calculated the BMD and BMDL of the cum Cd-A, mean cum Cd-B and leasq cum Cd-B for β2-MG uria using 5% and 10% of the benchmark response (BMR). Values of cumulative Cd exposures were log-transformed according to the previous study [4]. To find the best-fit model for BMD estimation, dichotomous Hill, gamma, logistic, probit, multistage, Weibull and quantal linear models were applied to the dose–response relationship for cum Cd-A, mean cum Cd-B and leasq cum Cd-B. Then, the BMD and BMDL values were calculated based on the best-fit model. The analysis of regression and curve estimation was performed with Benchmark Dose Software (version 3.1.2) available from the US EPA (Washington, DC, USA).

This study is based on previously published data [4] and does not involve any personal information. As such, we consider that it will not cause any detriment to the participants and no new informed consent is required.

## 3. Results

Table 1 shows the averages of cumulative cadmium exposure and cadmium concentrations corresponding to the entire working period of 40 or 30 yrs. The estimated average Cd-A was higher than the recent guidelines of ACGIH and SCOEL.

The responses with cum Cd-A, mean cum Cd-B and leasq cum Cd-B as the dose parameters are shown in Table 2a–c. These results except OR and P-values were shown in the previous report [4]. All categories except 5000–<1000 months nmol/L group of mean cum Cd-B showed significantly increased OR compared to the lowest control categories for cum Cd-A, mean cum Cd-B and leasq cum Cd-B. Furthermore, the odds ratio was clearly increased as the exposure level increased, indicating a strong dose–response relationship between renal tubular damage and cum Cd-A, mean cum Cd-B and leasq cum Cd-B. Thus, LOAEL for cum Cd-A was 691 yrs μg/m^3^, which corresponds to 17.3 or 23.0 μg/m^3^, assuming the whole working period to be 40 or 30 yrs. In terms of Cd-B, NOAEL (mean cumulative, 7122 months nmol/L) corresponded to 14.8 or 19.8 nmol/L (1.7 or 2.2 μg/L), and LOAEL (leasq cumulative, 8586 months nmol/L) was 17.9 or 23.9 nmol/L (2.0 or 2.7 μg/L).

**Figure 1:**
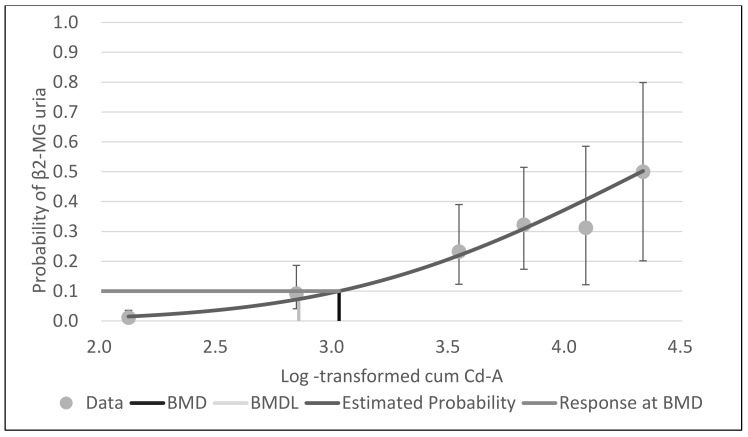
Probit model with BMR of 10% extra risk for the BMD and 95% lower confidence limit for the BMDL of log-transformed cum Cd-A for β2-MG uria.

**Figure 2:**
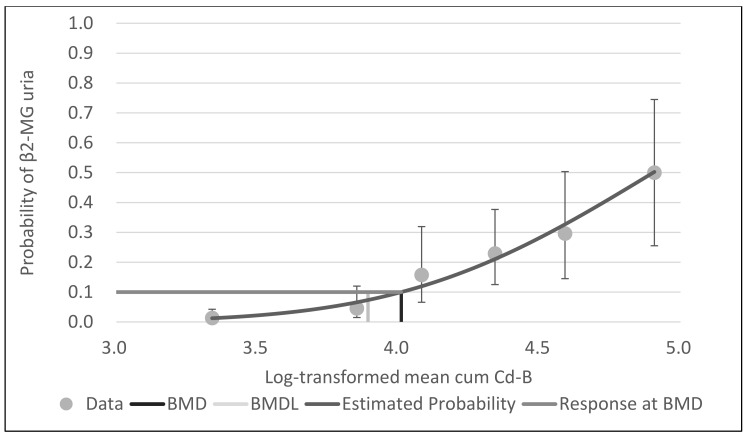
Probit model with BMR of 10% extra risk for the BMD and 95% lower confidence limit for the BMDL of log-transformed mean cum Cd-B for β2-MG uria.

**Figure 3:**
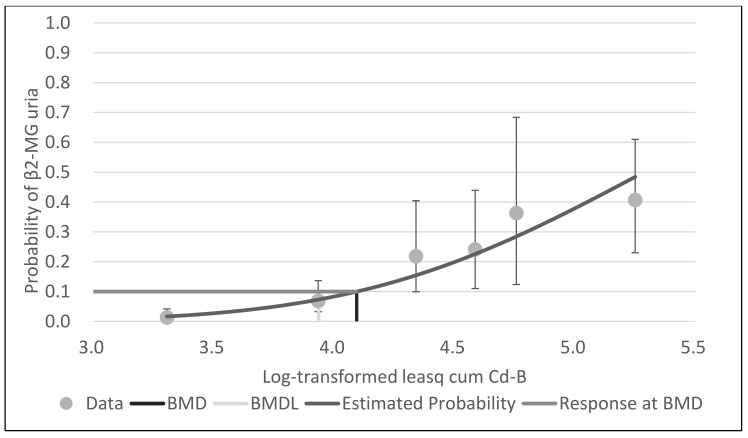
Probit model with BMR of 10% extra risk for the BMD and 95% lower confidence limit for the BMDL of log-transformed leasq cum Cd-B for β2-MG uria.

With BMDS software, the probit model was the best-fit model for all data for cum Cd-A, mean cum Cd-B and leasq cum Cd-B, based on Akaike’s information criteria. Figure 1, Figure 2 and Figure 3 show the dose–response curves obtained by probit models. These curves indicated a fine fit as an estimate of the actual dose–response relationship between cum Cd-A, mean cum Cd-B and leasq cum Cd-B and β2-MG uria.

Table 3 shows the BMD and BMDL of cum Cd-A, mean cum Cd-B and leasq cum Cd-B for β2-MG by the probit model of BMDS software. The BMDLs of cum Cd-A were 272.3 yrs µg/m^3^ (BMR5%) and 707.5 yrs µg/m^3^ (BMR10%). Assuming the whole working period to be 40 or 30 yrs, Cd-A would correspond to 6.8 or 9.1 µg/m^3^ (BMR5%) and 17.7 or 23.6 µg/m^3^ (BMR10%). The BMDLs of mean cum Cd-B were 3967.2 months nmol/L (BMR5%) and 7798.1 months nmol/L (BMR10%). Assuming the whole working period to be 40 or 30 yrs, Cd-B was estimated to be 8.3 or 11.0 nmol/L (BMR5%) and 16.2 or 21.7 nmol/L (BMR10%), corresponding to 0.9 or 1.2 µg/L (BMR5%) and 1.8 or 2.4 (BMR10%). The BMDLs of leasq mean Cd-B were 3588.6 months nmol/L (BMR5%) and 8616.3 months nmol/L (BMR10%). Assuming the whole working period as above, Cd-B was estimated to be 7.5 or 10.0 nmol/L (BMR5%) and 18.0 or 23.9 nmol/L (BMR10%), corresponding to 0.8 or 1.1 µg/L (BMR5%) and 2.0 or 2.7 µg/L (BMR10%).

## 4. Discussion

In the present study, we re-examined the previous data and estimated the BMD/BMDL of Cd for renal tubular damage. The concept of BMD/BMDL has been widely adopted for health risk assessment of various compounds. However, to our knowledge, the BMD/BMDL of Cd-A or Cd-B has not been estimated in terms of occupational exposure. Furthermore, it is also worth mentioning that we could compare several models for dose–response relationships between Cd exposure and renal effects by using BMDS software. The probit model adopted in the previous study [4] was the model that fit best to the data in the present study, further confirming the accuracy of the previous study [4]. With regard to urinary Cd, the BMD/BMDL was estimated as the threshold value of urinary Cd for renal tubular damage in 599 French, Swedish and US workers (451 men and 148 women, mean age: 45.4 years and mean exposure period: 18.8 years) who were employed in four nickel–cadmium battery plants [16]. By using the Hill model, the BMD_05_/BMDL_05_ of urinary Cd for abnormal urinary RBP and ß2-MG were estimated to be 5.1/3.0 and 9.6/5.9 µg/g cre in all workers, 12.6/6.6 and 12.2/5.5 µg/g cre in non-smokers, and 6.3/4.9 and 4.3/3.5 µg/g cre in smokers, respectively. In the present study, we estimated the NOAEL and LOAEL of Cd-A and Cd-B, which had not been determined in the previous publication [4]. The determination of NOAEL or LOAEL has been considered important for decision-making regarding the limit value to protect workers from harmful health effects from exposure to the substance used. Based on this study, the threshold level for renal damage seemed to be less than 20 µg/m^3^ for Cd-A and 2.0 µg/L for Cd-B.

As noted above, blood cadmium is a good indicator of internal dose after chronic exposure, with its biological half-life of 7–16 years [1]. Therefore, the balance between individual exposure and excretion may be an important factor for chronic exposure. In Table 3 in this study, the cumulative blood Cd was converted to blood concentrations, assuming that the exposure is constant and continued for 30 or 40 years, whereas we believe that the models including the elimination of accumulated cadmium may provide more useful information for the interpretation of the results for the cumulative blood Cd.

In recent years, the background Cd exposure is considered to have decreased. Therefore, we need to pay attention to the obtained data in view of the possibility that the renal outcome represents occupational and background exposure simultaneously about the earlier data. However, as there are limited data on Cd-A, we believe that the present data are still valuable for the occupational setting. On the other hand, Cd-B reflects the whole body burden of Cd. In view of the low background exposure in recent years, higher Cd-B depends on the amount of occupational exposure, emphasizing the importance of countermeasures against it. In terms of outcome, we adopted tubular damage as reflecting the early change of Cd exposure. For a further long-term health outcome, increased mortality was associated with cadmium exposure in longitudinal studies in the general population [17,18]. Therefore, we believe that further studies in the worker population are necessary.

Several studies have previously investigated the dose–response relationship between cumulative Cd-A and renal damage in the working environment. Ellis et al. [19] evaluated the relationship between chronic cadmium exposure and the cadmium body burden in 82 industrially exposed workers. For the currently employed workers, a significant correlation was observed between the cumulative Cd-A and liver cadmium burden, measured by the in vivo prompt-gamma neutron activation technique. Furthermore, whenever the worker’s liver burden exceeded 40 ppm and the exposure index exceeded 400–500 yrs μg/m^3^, there was evidence of renal abnormalities (usually elevated urinary β2-MG). The percentage of workers with renal abnormalities was found to increase as the cumulative Cd exposure index increased. Mason et al. [5] investigated renal damage by cumulative Cd exposure in 75 exposed male workers and an equal number of referents matched for age, sex, and employment status. Significant increases in the urinary excretion of albumin, retinol binding protein (RBP), β2-MG, N-acetylglucosaminidase (NAG), alkaline phosphatase, gamma-glutamyl transferase and significant decreases in the renal reabsorption of calcium, urate, phosphate and glomerular filtration rate were found in the exposed group compared with the referent group. Furthermore, by two phase linear regression, inflection points signifying a threshold level of cumulative Cd-A were identified for the dose–effect relationship concerning those renal markers. Urinary total protein, RBP, albumin, and β2-MG gave similar inflection points at a cumulative Cd exposure of about 1100 yrs µg/m^3^. The 95% lower limit of inflection points was 509 yrs µg/m^3^ for urinary β2-MG and 636 yrs µg/m^3^ for RBP. Simple dose–response analysis of the exposed group showed a greatly increased incidence of tubular proteinuria when the cumulative Cd exposure was greater than 1000 yrs µg/m^3^. Thus, these results indicated that the threshold level of cumulative Cd-A was 510–1100 yrs µg/m^3^, corresponding to a 40-year exposure of 12.8–27.5 µg/m^3^. Thus, we believe that the results of the present study and those of previous studies complement each other and add important findings on the health effects of Cd-A for occupational exposure.

With respect to Cd-B, several studies have shown a higher incidence of renal dysfunction in exposed workers. Chia et al. [6] studied Cd-induced renal tubular effects in 65 female workers in a factory manufacturing nickel–cadmium batteries. Urinary NAG and urinary β2-MG showed a strong positive correlation with Cd-B. Abnormal urinary β2-MG was detected in only 15.4% of the workers, half of whom had B-Cd above 10 μg/L. The age adjusted mean urinary NAG showed a rise from 1 μg/L of Cd-B followed by a plateau between Cd-B of 3–10 μg/L. No significant rise in mean urinary excretion in β2-MG was seen until Cd-B exceeded 10 μg/L. Thus, NAG and β2-MG increased appreciably when the B-Cd was greater than 10 μg/L. Bernard et al. [7] investigated 58 workers exposed to Cd in a non-ferrous smelter and from 58 age matched referents. In terms of urinary tubular markers, the prevalences of increased urinary β2-MG, RBP, albumin and NAG were increased only in workers with Cd-B higher than 10 μg/L. The present study showed that the BMDL and NOAEL of Cd-B were 1–2 μg/L when assuming 40 years of work, which was even lower than those threshold levels noted in previous studies [6,7]. This is also considered to be due to the fact that the number of subjects was 3–6 times larger than those in the previous studies. Furthermore, the β2-MG values were determined as an average of the available measurements (1–8 times) to avoid single laboratory findings above the critical level influencing the results. Therefore, we believe that the intra-individual variability has been substantially corrected for, and that the determination of the renal effects is improved due to this better reliability of the results compared to those in a cross-sectional survey based on a single measurement. We also believe that this is a major reason why the dose–response relationship was found in a clear form, as shown in Figure 2.

In terms of the allowable limit for occupational exposure, ACGIH reviewed the TLV of Cd-A [8] and BEIs of Cd-B and urinary Cd [9]. It was concluded that the TLV and BEIs should be maintained as the previous values of 10 µg/m^3^ (total particulate), 2 µg/m^3^ (respirable particulate fraction) (Cd-A), 5 µg/L (Cd-B) and 5 µg/g cre (urinary Cd). In particular, for Cd-B, ACGIH decided to retain the BEI of Cd-B because there was no new information after the previous study [4] which was recognized as the essential information. On the other hand, a recent re-evaluation report in the EU set the 8-h TWA of Cd-A as 1 µg/m^3^ (inhalable fraction) and the BEV of urinary Cd at 2 μg/g cre, without setting the BEV of Cd-B due to the limited information [10]. Assuming a working period of 40 years, the results of LOAEL, BMDL_05_, and BMDL_10_ for Cd-A in this study were generally in agreement with the guideline of ACGIH, but were higher than the EU guideline level. On the other hand, the results for Cd-B were lower than the guideline of ACGIH, suggesting the need for reconsideration of the guidelines in the future.

One limitation of the present study was the lack of information on other factors, which thus could not be corrected for in this study, especially smoking. The analysis of publicly available biomonitoring data showed that B-Cd and urinary Cd are higher in cigarette smokers [20]. Therefore, individual susceptibility to the renal effects of cadmium and potentially different effects on levels could not be clarified in detail. Descriptive data of the subgroup with evidence of renal tubular damage would be extremely valuable, even though this has been reported previously.

## 5. Conclusions

The present study revealed essential information for health risk assessment, such as NOAEL and BMDL for Cd exposure. In addition, it was suggested that the reference concentration of Cd-B, for which less information was available, was lower than the previously assumed one. In the ongoing discussion on the exposure limits for Cd, this study provides new valuable information to enhance the reliability of limit values, thereby making a significant contribution to preventing adverse health effects of Cd in exposed workers.

## Figures and Tables

**Table 1:** Averages of cumulative cadmium exposure and cadmium concentrations corresponding to the entire working period of 40 or 30 yrs.

		Working Period	
Cd Exposure	Average Dose	40 yrs	30 yrs
Cum Cd-A	1929 yrs μg/m^3^	48.2 μg/m^3^	64.3 μg/m^3^
Mean cum Cd-B	11350 months nmol/L	23.6 nmol/L (2.7 µg/L)	31.5 nmol/L (3.5 µg/L)
Leasq cum Cd-B	19878 months nmol/L	41.4 nmol/L (4.7 µg/L)	55.2 nmol/L (6.2 µg/L)

**Table 2:** (**a**) Dose–response relationship using cumulative air-cadmium as the dose-indicator. (**b**) Dose–response relationship using mean cumulative blood-cadmium as the dose-indicator. (**c**) Dose–response relationship using cumulative blood-cadmium as the dose-indicator (least-squares estimate).

(**a**)						
**Cum Cd-A** **(yrs μg/m^3^)**	**Average Dose** **(yrs μg/m^3^)**	**N**	**β2-MG Uria**	**Response Rate**	**OR (95% CI)**	***p***
<359	131	264	3	1.1%		
359–<1710	691	76	7	9.2%	8.8 (2.2, 35.0)	0.002
1710–<4578	3460	43	10	23.3%	26.4 (6.9, 100.7)	<0.001
4578–<9458	6581	31	10	32.3%	41.4 (10.6, 162.2)	<0.001
9458–<15,000	12,156	16	5	31.3%	39.5 (8.4, 186.9)	<0.001
15,000+	21,431	10	5	50.0%	87.0 (16.2, 468.1)	<0.001
(**b**)						
**Mean Cum Cd-B** **(Months nmol/L)**	**Average Dose** **(Months nmol/L)**	**N**	**β2-MG Uria**	**Response Rate**	**OR (95% CI)**	***p***
<5000	2185	221	3	1.4%		
5000–<10,000	7122	87	4	4.6%	3.5 (0.8, 16.0)	0.106
10,000–<15,000	12,077	38	6	15.8%	13.6 (3.2, 57.2)	<0.001
15,000–<30,000	21,985	48	11	22.9%	21.6 (5.8, 81.1)	<0.001
30,000–<60,000	38,947	27	8	29.6%	30.6 (7.5, 125.0)	<0.001
60,000+	80,738	16	8	50.0%	72.7 (16.2, 326.6)	<0.001
(**c**)						
**Leasq Cum Cd-B** **(Months nmol/L)**	**Average Dose** **(Months nmol/L)**	**N**	**β2-MG Uria**	**Response Rate**	**OR (95% CI)**	***p***
<5000	2019	223	3	1.3%		
5000–<10,000	8586	115	8	7.0%	5.5 (1.4, 21.1)	0.013
10,000–<15000	21,870	32	7	21.9%	20.5 (5.0, 84.5)	<0.001
15,000–<30,000	38,527	29	7	24.1%	23.3 (5.6, 96.7)	<0.001
30,000–<60,000	57,087	11	4	36.4%	41.9 (7.8, 223.8)	<0.001
60,000+	177,928	27	11	40.7%	50.4 (12.8, 199.2)	<0.001

OR: Odds ratio, 95% CI: 95% confidence intervals.

**Table 3:** Benchmark doses of cumulative cadmium exposure and cadmium concentrations corresponding to the entire working period of 40 or 30 yrs.

Cd exposure	BMR5%		BMR10%	
	BMD	BMDL	BMD	BMDL
Cum Cd-A (yrs µg/m^3^)	451.2	272.3	1055.2	707.5
Cd-A (µg/m^3^, 40 yrs)	11.3	6.8	26.4	17.7
Cd-A (µg/m^3^, 30 yrs)	15.0	9.1	35.2	23.6
Mean cum Cd-B (months nmol/L)	5712.9	3967.2	10233.6	7798.1
Cd-B [nmol/L (µg/L), 40 yrs]	11.9 (1.3)	8.3 (0.9)	21.3 (2.4)	16.2 (1.8)
Cd-B [nmol/L (µg/L), 30 yrs]	15.9 (1.8)	11.0 (1.2)	28.4 (3.2)	21.7 (2.4)
Leasq cum Cd-B (months nmol/L)	5688.9	3588.6	12399.2	8616.3
Cd-B [nmol/L (µg/L), 40 yrs]	11.9 (1.3)	7.5 (0.8)	25.8 (2.9)	18.0 (2.0)
Cd-B [nmol/L (µg/L), 30 yrs]	15.8 (1.8)	10.0 (1.1)	34.4 (3.9)	23.9 (2.7)

## Data Availability

The data that support the findings of this study are available from the corresponding author upon reasonable request.
